# Janus kinase inhibitor and rituximab in rheumatoid arthritis: is it a safe combination?

**DOI:** 10.1093/rap/rkag042

**Published:** 2026-03-31

**Authors:** Hadi Rabee, Arti Mahto

**Affiliations:** Faculty of Life Sciences & Medicine, King’s College London, London, UK; Rheumatology Department, King’s College Hospital NHS Trust, London, UK

Key message

- Two-year patient follow-up shows rituximab plus filgotinib maintains low disease activity, and is safe and well-tolerated in refractory RA.

Dear Editor, We are writing as part of the same clinical team that previously reported the case titled ‘Combining B cell depletion and JAKi for difficult-to-treat rheumatoid arthritis’ (Lee S.Y. *et al*., Rheumatology Advances in Practice, 2023) [1]. In that report, we described a patient with refractory seropositive RA who achieved a marked and sustained improvement following combination therapy with rituximab and filgotinib (started at a dose of 200 mg once daily, and then subsequently reduced to 100 mg once daily), after multiple prior biologic and targeted synthetic DMARDs (bDMARDs and tsDMARDs) had failed.

Since publication, the patient has remained under our ongoing care, allowing us to observe the longer-term safety and efficacy of this therapeutic approach. We wish to provide an update on the patient’s clinical course over an extended follow-up period of >2 years, during which the combination regimen has continued to maintain low disease activity without new adverse events. This correspondence aims to contribute additional longitudinal evidence regarding the potential role of combined B cell depletion and Janus kinase (JAK) inhibition in managing difficult-to-treat RA, an area where real-world data remain limited.

Since the initial report, the patient has remained under regular rheumatology follow-up with clinical reviews every 3–4 months. Disease activity has been consistently low, with a 28-joint DAS with CRP in the low disease activity or remission domains, with normalisation of the inflammatory markers and successful weaning down of the steroid, as shown in Fig. 1. Upon reviewing the antibody titres, we observed a decrease in RF and CCP antibody levels following the combination treatment (RF is 62 IU/ml from 864 IU/ml pre-treatment and CCP is 66 U/ml from >340 U/ml pre-treatment). The patient continues combination therapy with rituximab (1 g × 2 doses, every 6 months) and reduced-dose filgotinib 100 mg once daily, alongside standard adjunctive care including calcium and vitamin D. No corticosteroid use has been required for >15 months.

**Figure 1:**
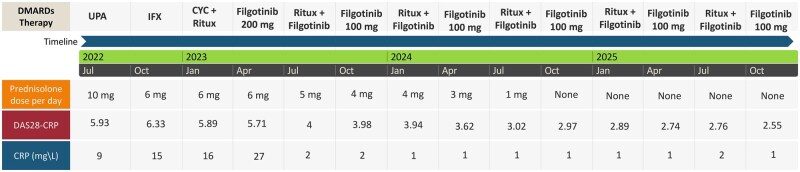
The timeline of the DMARD therapy and the disease characteristics of the patient. UPA: upadacitinib; IFX: infliximab; CYC: cyclophosphamide; Ritux: rituximab

Importantly, the combination has remained well tolerated with no serious infections, cytopenias or hepatic abnormalities observed. Routine monitoring of liver enzymes, lipid profile and full blood count has remained within normal limits. The patient reports a substantial improvement in quality of life and functional ability, reflected in a sustained reduction in his HAQ Disability Index score and full return to his baseline function. No infusion-related or JAK inhibitor–associated adverse events have been encountered to date.

JAK inhibitors and B cell depletion therapy by rituximab have been approved for the treatment of severe RA patients who failed to achieve a treatment target (i.e. failure to respond) or when there is a contraindication to TNF-α inhibitors [2–4]. The concept of dual biologic therapy to target distinct inflammatory pathways has gained significant attention as a potential solution for the unmet clinical needs of patients with refractory disease. Many bDMARD combinations have been looked at in a meta-analysis, which showed good efficacy outcomes, but obviously with a concern for adverse safety events for most tested combinations in this analysis [5]. The combination of JAK inhibitor (mainly tofacitinib and baricitinib) and rituximab has been reported in the treatment of anti-melanoma differentiation-associated protein 5 antibody-positive dermatomyositis–associated interstitial lung disease with promising outcomes, albeit with increased incidence of infective complications in a significant proportion of patients [6]. To our knowledge, this is the first case report of using a combination of JAK inhibitor (filgotinib) and rituximab for the treatment of difficult-to-treat RA with good outcome and no reported adverse events (including increased risk of infection) over a prolonged period of time.

A patient-centred treatment approach is particularly important when considering riskier treatment strategies, with each treatment consultation necessitating a thorough discussion of the potential risks and benefits. This long-term follow-up supports the potential durability and tolerability of combined B cell depletion and JAK inhibition in difficult-to-treat RA, warranting further evaluation in larger studies.

## Data Availability

All relevant patient information is included in the article with patient consent.
